# Activated PI3K*δ* syndrome 1 mimicking systemic lupus erythematosus and secondary Sjögren's syndrome-like phenotype without recurrent infections: A case report

**DOI:** 10.3389/fped.2022.1077324

**Published:** 2022-12-20

**Authors:** Jing Yin, Jijun Ma, Jingyue Xia, Yang Cao, Chongwei Li

**Affiliations:** Department of Rheumatology & Immunology, Tianjin Children's Hospital, Tianjin University, Tianjin, China

**Keywords:** activated phosphoinositide 3-kinase-*δ* syndrome, PIK3CD, p110*δ*, autoimmune, systemic lupus erythematosus, Sjögren's syndrome

## Abstract

Activated phosphoinositide 3-kinase-*δ* syndrome 1 (APDS1) is a combined immunodeficiency caused by a heterozygous gain-of-function mutation in *PIK3CD*, encoding the p110*δ* catalytic subunit of phosphoinositide 3-kinase *δ* (PI3K*δ*). APDS1 is characterized by recurrent sinopulmonary infections, leading to airway damage, chronic herpes viremia, lymphoproliferation, and autoimmune and inflammatory diseases. Several cases of systemic lupus erythematosus (SLE) have been reported in APDS1; however, Sjögren's syndrome (SS) or an SS-like phenotype is rarely described in patients with APDS1. In this study, we report a 4-year-old girl with APDS1 who did not experience recurrent sinopulmonary infections and chronic viremia but presented with cytopenia, proteinuria, hypocomplementemia, and positive antinuclear antibodies that met the classification criteria for SLE. Additionally, the patient also mimicked a secondary SS-like phenotype based on recurrent parotitis and labial salivary gland biopsy. The patient achieved remission after treatment with sirolimus and immunosuppressive therapy. This case report enriches the clinical phenotype of APDS1 and provides a reference for the diagnosis and therapy of patients with APDS1.

## Introduction

Phosphoinositide 3-kinases (PI3Ks) are a family of enzymes discovered by Whitman et al. in 1985 (1). Class IA PI3Ks are heterodimers and composed of a catalytic subunit (p110*α*, p110*β*, p110*γ*, and p110*δ*) and a regulatory subunit (p85*α*, p85*β*, p55*α*, p55*γ*, and p50*α*) (2). After receptor engagement, PI3K phosphorylates phosphatidylinositol 4,5-bisphosphate (PIP2) to generate phosphatidylinositol-3,4,5-trisphosphate (PIP3) as a secondary messenger to recruit kinases PDK1 and AKT (3, 4). AKT signaling stimulates the mechanistic target of rapamycin (mTOR) to directly phosphorylate p70s6 kinase (p70s6k), an mTOR substrate that plays an important role in protein synthesis (4). The PI3K/AKT/mTOR signaling axis is vital in regulating various cellular functions, including cellular metabolism, gene expression, and post-translational regulation of protein and organelle functions. The important roles of these proteins in the immune system, especially in T and B lymphocytes, have been widely described (5, 6).

Gain-of-function (GOF) mutations in *PIK3CD*, which encodes the p110*δ* catalytic subunit, result in a dramatic increase in p110*δ* activity, with increased AKT phosphorylation and mTOR activity. These activating mutations, *p*.E1021K being the commonest, lead to an autosomal-dominant primary immune deficiency called “activated phosphoinositide 3-kinase-*δ* syndrome 1 (APDS1)” (7, 8). Meanwhile, germline mutations in *PIK3R1*, which encodes the inhibitory subunits (p85*α*, p55*α*, and p50*α*), also can result in an increased phosphoinositide 3-kinase-*δ* (PI3k*δ*) activity that is termed APDS2, sharing many clinical features with APDS1. The main clinical and immunological characteristics of APDS1 are recurrent sinopulmonary infections leading to airway damage, chronic Epstein–Barr virus (EBV) and/or cytomegalovirus (CMV) viremia, benign lymphoproliferation, progressive lymphopenia, and a hyper-immunoglobulin M (IgM) phenotype. In addition, autoimmune or autoinflammatory diseases, including systemic lupus erythematosus (SLE), autoimmune hemolytic anemia, autoimmune thrombocytopenia, chronic arthritis, and inflammatory bowel disease, have also been reported (9, 10). However, reports of Sjögren's syndrome (SS) and SS-like phenotypes are rare in APDS1. In this case report, we describe a young girl with a GOF mutation in *PIK3CD* who mimicked SLE and secondary SS-like manifestations without recurrent infections.

## Case description

A 4-year-old girl was admitted to our hospital in September 2017 with intermittent fever for 7 days and intermittent gross hematuria for 3 days. No other unpleasant symptoms were noted. She had a history of splenomegaly when she was 1 year old; however, this finding was not followed up, and she had not been re-examined. She had 7–8 episodes of fever with thrombocytopenia since the age of one-and-a-half year, and her platelet count returned to normal after the temperature decreased to the normal range. She had no history of recurrent or severe infections, including pneumonia, media otitis, and enteritis. She had a healthy younger sister.

**Figure 1:**
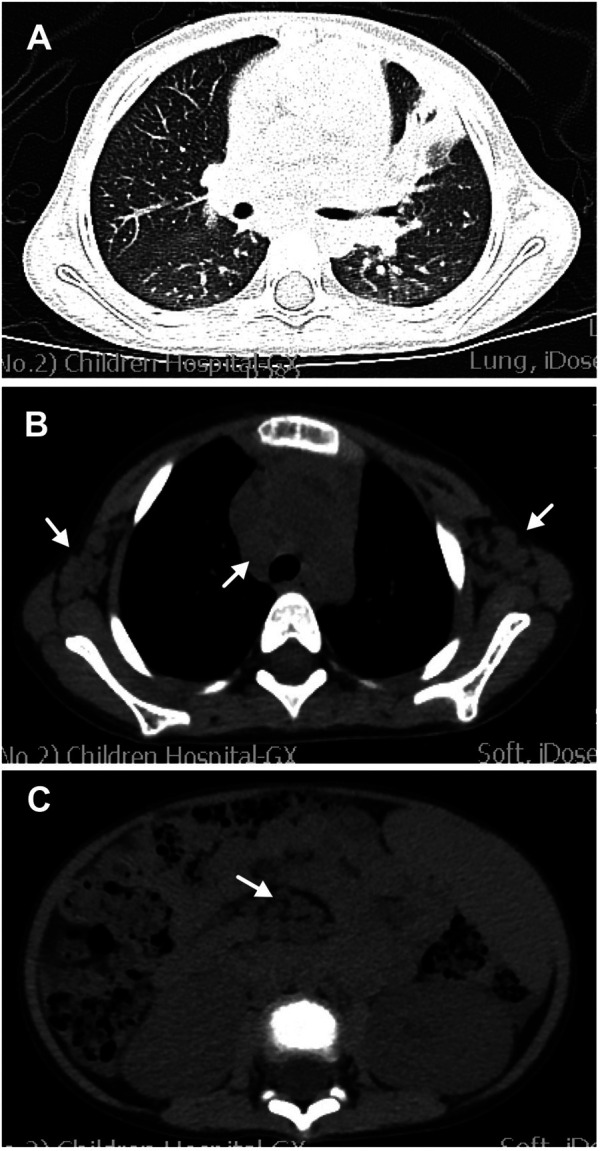
Computed tomography (CT) scanning of the chest and abdomen. (**A**) Lung CT scan showing scattered ground-glass opacity in both lungs and consolidation in the left lung. (**B**) Lung CT scan showing enlargement of multiple lymph nodes on the axillae and mediastinum (white arrows). (**C**) Abdominal CT scan showing enlargement of multiple lymph nodes (white arrows) and enlarged spleen and kidney.

Physical examination revealed enlarged lymph nodes and spleen (4 cm below the left costal margin). The spleen continued to grow within the first few days of hospitalization. The liver was not palpable. Cardiopulmonary examination results were unremarkable. No rash was noted. The preliminary complete blood count revealed mild anemia with a hemoglobin count of 98 g/L, a white blood cell count (WBC) of 2.57 × 10^9^/L, of which 52% were neutrophils and 40% were lymphocytes, and a platelet count of 113 × 10^9^/L. The C-reactive protein (CRP) was < 2.5 mg/L (0–8). Urinalysis revealed proteinuria and microscopic hematuria. Moreover, the 24-h quantitative urine protein was 1,213.7 mg (0–150). The hepatorenal function was normal. Prothrombin time (PT) was 12.2 s (10.6–16), activated partial thromboplastin time (APTT) was 129.6 s (20–40), and the fibrinogen level was 4.033 g/L (1.8–4.0). Both anti-EBV and anti-CMV IgM tests were negative. The patient tested positive for anti-EBV immunoglobulin G (IgG) and negative for the EBV load. Ultrasound showed diffuse renal lesions and splenomegaly. Computed tomography (CT) scanning showed scattered ground-glass opacity in both lungs and consolidation in the left lung (Figure 1A), multiple lymphadenopathies on the axillae, mediastinum, groins, and the root part of the mesentery, and enlarged kidneys and spleen (Figures 1B,C).

**Figure 2:**
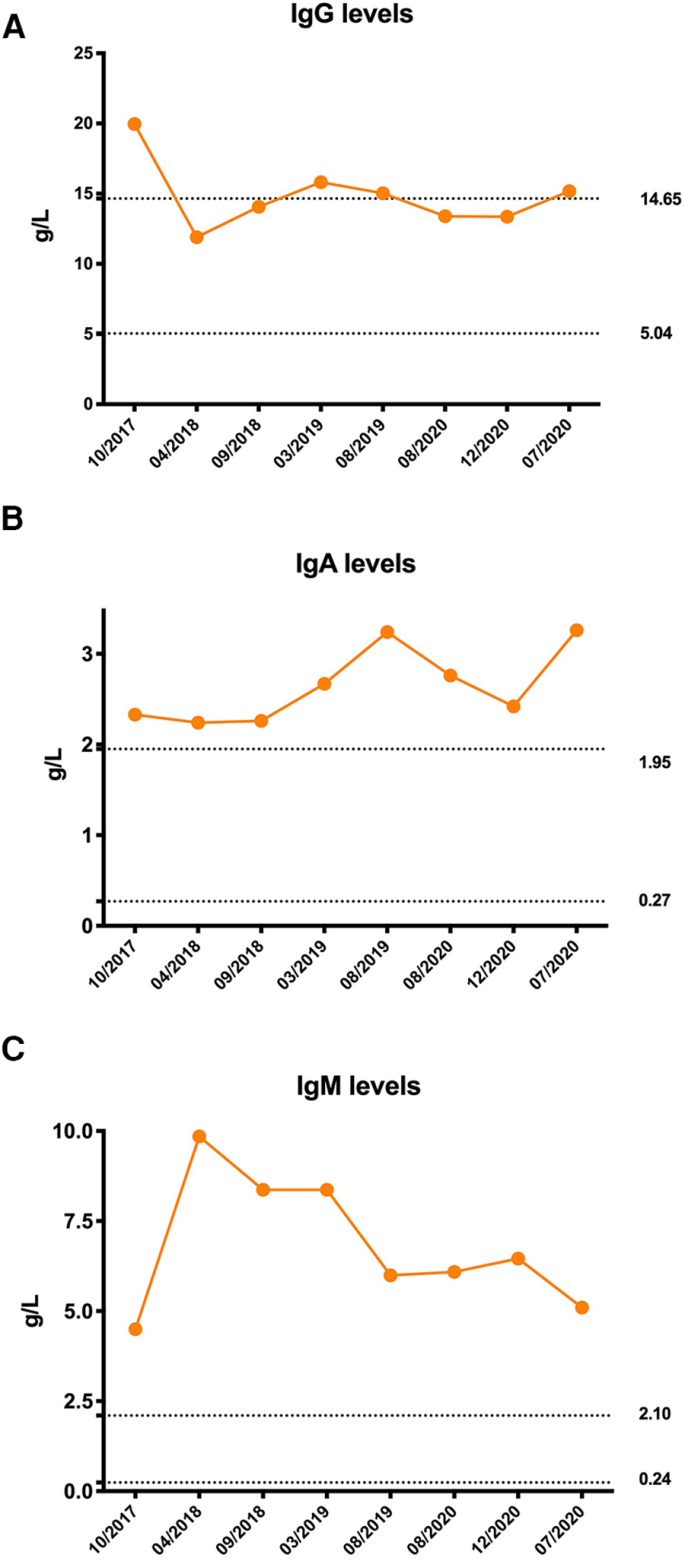
Immunoglobulin levels of the patient. (**A**) Initial serum immunoglobulin G (IgG) level was significantly elevated and remained at the upper limit of normal after treatment. (**B**) Serum immunoglobulin A (IgA) levels were persistently higher than normal. (**C**) Serum immunoglobulin M (IgM) levels were persistently higher than normal. The dotted line was the normal reference value.

Owing to coagulation abnormalities, fresh frozen plasma was administered to the patient. However, the APTT was >180 s following treatment, which is considerably longer than that before treatment. Therefore, a cryoprecipitate, mainly containing factor VIII and fibrinogen, was transfused into the patient, and her APTT gradually returned to normal. On the third day of hospitalization, repeated blood tests showed a further decrease in hemoglobin level and WBC count, and the platelet count decreased to 39 × 10^9^/L. Her serum complement 3 (C3) and complement 4 (C4) levels were 0.27 g/L (0.9–1.8) and 0.04 g/L (0.1–0.4), respectively. Her direct and indirect Coombs tests were negative, as was the case for antiphospholipid antibodies. Antinuclear antibodies were positive at a titer of 1:1000. High titers of antibodies against double-stranded DNA, nucleosomes, and ribosomes were also found. The serum immunoglobulin levels were all higher than normal: IgG 19.96 g/L (5.04–14.65) (Figure 2A), immunoglobulin A (IgA) 2.33 g/L (0.27–1.95) (Figure 2B), and IgM 4.5 g/L (0.24–2.1) (Figure 2C). Flow cytometry analyses showed that the percentages of CD3^+^ T, CD4^+^ T, CD8^+^ T, CD19^+^ B, and NK cell subsets were all within the normal range. Moreover, clinical manifestations and laboratory findings met the 2012 Systemic Lupus International Collaborating Clinics (SLICC) classification criteria. Therefore, the patient was started on prednisone and showed a good response.

However, several issues were noticed regarding the diagnosis of this patient. The patient was only 4 years old, and she had splenomegaly and several episodes of transient thrombocytopenia since the age of 1 year, which may have been the onset of the disease. Therefore, monogenetic SLE was suspected. Therefore, whole-exome and Sanger sequencing analyses were performed, which revealed a heterozygous missense mutation in gene *PIK3CD* (c.3061G < A, *p*.E1021K) encoding the p110*δ* catalytic subunit, an autosomal-dominant GOF mutation that has been previously reported (7, 8). Her parents were wild-type. No other disease-relevant mutations were found. Therefore, the patient was diagnosed with APDS1. B-cell immunophenotyping analysis showed no elevation in transitional B-cell counts. Treatment with sirolimus (rapamycin) was initiated, and prednisone was gradually tapered. Her lymph nodes and spleen returned to almost normal size, and proteinuria disappeared after treatment. In 2018, she developed swelling and pain in the right parotid gland that persisted for 1 month. In March 2020, prednisone was discontinued, and only sirolimus was administered at 1 mg/day.

**Figure 3:**
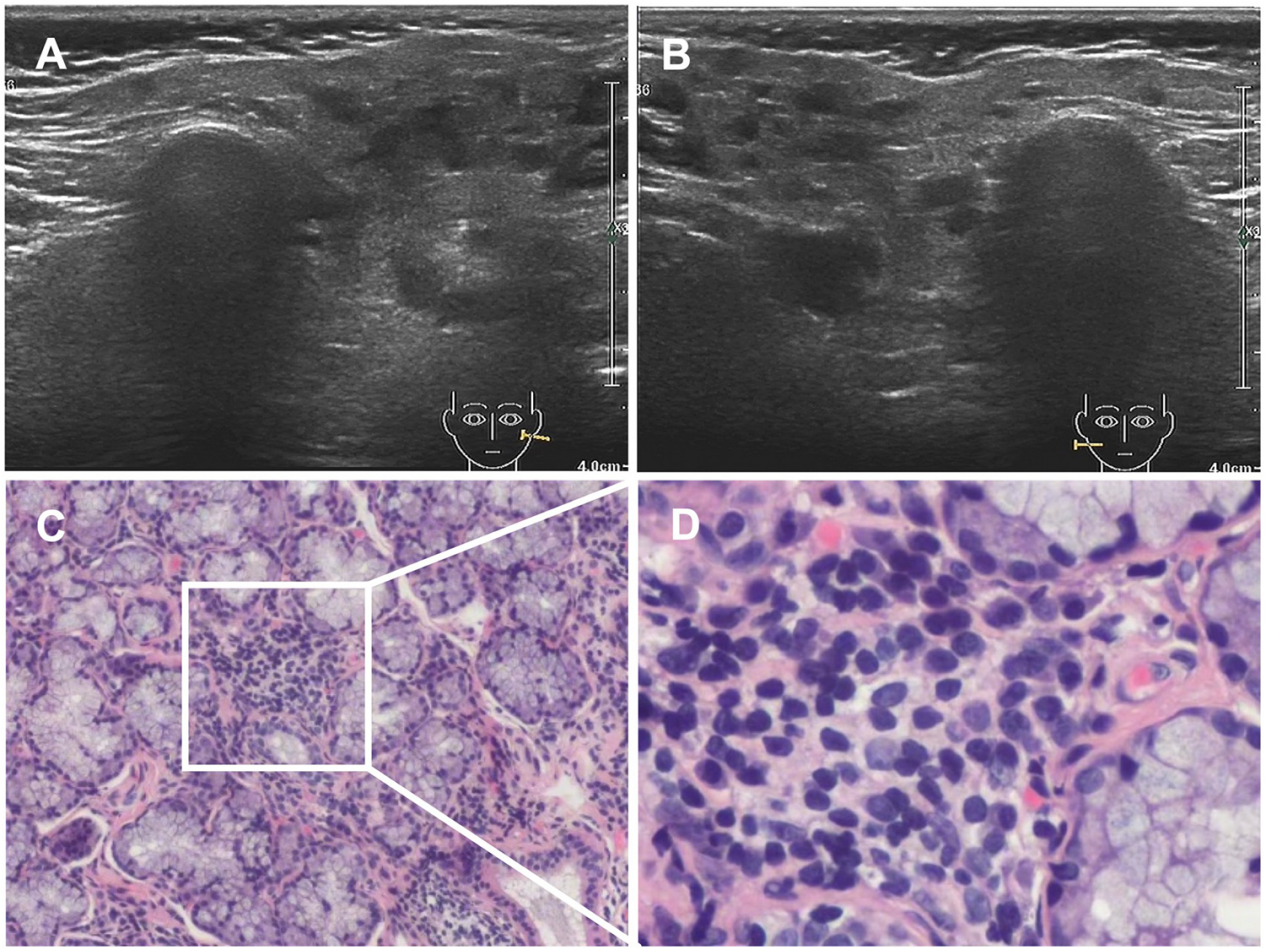
Results of ultrasound and LSGB. (**A**,**B**) Ultrasound scan showing diffuse bilateral parotid lesions. (**C,D**) LSGB revealing multifocal lymphocytic infiltration between lobules, dilated interlobular ducts, and occasional destruction of the gland. LSGB, labial salivary gland biopsy.

**Figure 4:**
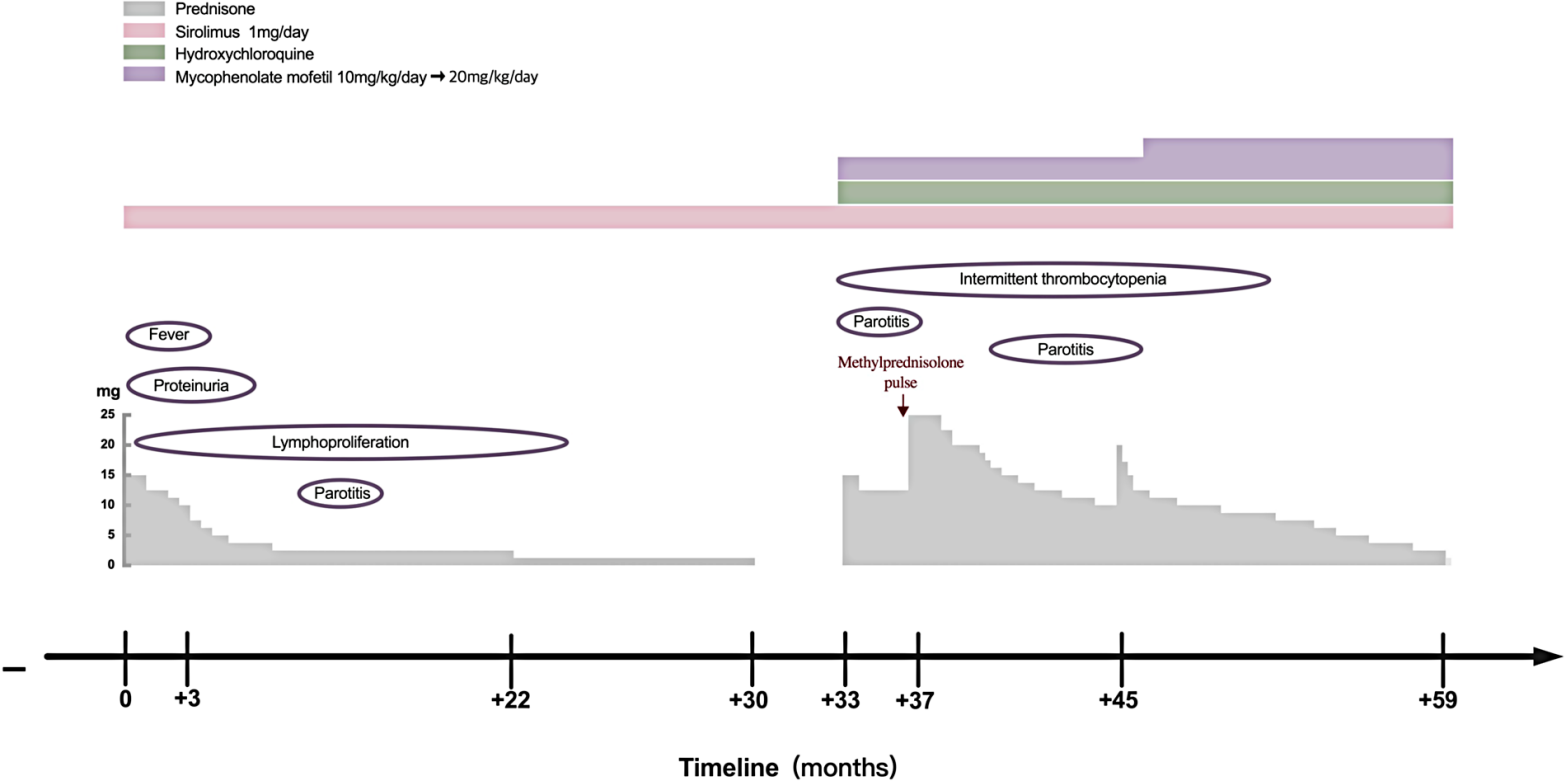
Clinical course of the patient with APDS1. APDS1, activated phosphoinositide 3-kinase-*δ* syndrome 1.

In June 2020, she presented with a swollen and painful parotid gland and developed mild thrombocytopenia (platelet count of 76 × 10^9^/L) without bleeding manifestation. No pathogens were found. Ultrasound showed right parotid enlargement and diffused bilateral parotid lesions (Figures 3A,B). Labial salivary gland biopsy (LSGB) revealed multifocal lymphocytic infiltration between the lobules, dilated interlobular ducts, and occasional destruction of the gland (Figures 3C,D). Therefore, in addition to sirolimus, prednisone, mycophenolate mofetil (MMF), and hydroxychloroquine (HCQ) were administered (Figure 4). Her parotitis improved, and the platelet count recovered. However, the platelet count decreased when prednisone was tapered. The patient received a large dose of IVIG (2 g/kg), after which she developed intense headaches, which were considered an adverse reaction to IVIG and relieved immediately after a dose of mannitol. Her head CT and magnetic resonance imaging revealed no abnormalities. No pathogens were found in the cerebrospinal fluid. In October 2020, thrombocytopenia reoccurred but was resolved following intravenous methylprednisolone pulse therapy. Intermittent bilateral parotid pain recurred in March 2021 and was not relieved until the prednisone dosage was increased to 20 mg/day in July 2021 (Figure 4). The patient remained stable until the paper was written, and prednisone was reduced to 2.5 mg/day.

## Discussion

Unlike other subunits that are ubiquitously expressed in all tissues, p110*δ* is restricted to the immune cells (11). Patients with APDS1 display clinical features of both immune deficiency and dysregulation. The most common T lymphocyte immunophenotype of this disorder is progressive lymphopenia, with a more prominent reduction in CD4^+^ T cells than in CD8^+^ T cells. T lymphocytes from patients with APDS1 show increased PI3K/mTOR activity and increased glucose uptake, which results in the activation of naïve T lymphocytes. Overactivation of PI3K/mTOR signaling depletes naïve T cell pools and promotes senescence and activation-induced death of T lymphocytes instead of proliferation and conversion to functional memory T cells. PI3K signaling is essential for many aspects of B-cell biology. Most patients with APDS1 had elevated levels of transitional B cells and decreased switched memory B cells, which resulted in a hyper-IgM phenotype (12). Consequently, patients with APDS1 typically present with recurrent sinopulmonary infections, a predisposition to chronic EBV and/or CMV viremia, and a poor response to vaccination (11). In several cohort studies, more than 95% of patients with APDS1 had recurrent or severe respiratory infections with persistent herpes virus infections (10, 13, 14). However, in our study, the patient had neither recurrent nor severe sinopulmonary infections nor persistent viremia, which could be attributed to her atypical immunophenotype presentation of APDS1. The patient did not have persistent lymphopenia or hyper-IgM phenotype. Although her serum IgM level was significantly elevated, her serum IgG and IgA levels were also elevated (Figures 2A–C).

Benign lymphoproliferation manifesting as lymphadenopathy, splenomegaly, hepatomegaly, or focal nodular lymphoid hyperplasia is another common clinical feature reported in 75% of patients with APDS1 in a large-cohort study (10). In addition, patients with APDS1 show increased susceptibility to B-cell lymphoma (15). Consistent with these reports, lymphadenopathy and splenomegaly were the prominent signs in our patient. Moreover, she developed splenomegaly at the age of 1 year, which was an important clue suggesting a genetic disorder. However, to date, no signs of lymphoma have been identified in the patient based on the routine examinations during the follow-up.

The aberrant activation of PI3Ks plays a key role in the pathogenesis of autoimmune and inflammatory diseases. Studies of humans and mice with germline *PIK3CD* GOF mutations showed that overactivated PI3K*δ* broke the tolerance of B cells and increased the positive selection of autoreactive B cells (16). Several cases of SLE have been reported in patients with APDS1 (9, 17) and APDS2 (18). However, SS-related manifestations of this disorder have been rarely described. The four previously reported patients with APDS1 and the SLE phenotype carried the same mutation (*p*.E1021K) and presented with benign lymphoproliferation, recurrent respiratory infections, and chronic herpes viremia. Two of the four cases had a typical hyper-IgM phenotype, whereas the other two had elevated IgM and IgG levels that were either normal or mildly increased (Table 1). APDS2 phenocopy APDS1, a case of a reported patient with APDS2 exhibited clinical features similar to the four patients with APDS1, but she developed lymphoma, which may be related to her age (Table 1). Our patient also carried the *p*.E1021K mutation and exhibited lymphoproliferation; however, she had no history of recurrent infections but had autoimmune disorders. Moreover, she mimicked SLE and the clinical features of the SS-like phenotype (Table 1). Unlike adult SS, juvenile SS rarely presents with symptoms of dry eyes and mouth. Parotitis is a more common manifestation in juvenile SS. Major salivary gland ultrasonography plays an important role in juvenile SS (19). Meanwhile, parotitis may also be lymphoproliferation, but it is more likely due to an SS-related autoimmune phenomenon based on the pathological findings. The absence of SS-specific antibodies (anti-SSB antibodies) and SS-associated antibodies (anti-SSA antibodies) suggests that SS-like features were secondary to SLE rather than an independent autoimmune phenotype in this patient.

**Table 1:** Clinical features of APDS patients with SLE phenotype.

	P1 (our patient)	P2	P3 (older brother of P2)	P4	P5	P6
Age of onset (years)	1	2	2	3	9	–
Age at diagnosis (years)	4	15	15	10	15	19
Gender (F/M)	F	M	M	M	M	F
Mutation gene	*PIK3CD*	*PIK3CD*	*PIK3CD*	*PIK3CD*	*PIK3CD*	*PI3KR1*
Mutation position	*p*.E1021K	*p*.E1021K	*p*.E1021K	*p*.E1021K	*p*.E1021K	Splice acceptor site at c.1300-2
IgM	Increased	Increased	Increased	Increased	Increased	Increased
IgG	Increased	Decreased	Decreased	Mildly increased	Normal	Normal
IgA	Increased	—	—	—	Normal	Decreased
CD4:CD8 ratio inversion	No	Yes	Yes	Yes	—	Yes
Recurrent respiratory infections	No	Yes	Yes	Yes	Yes	Yes
Chronic herpes viremia	No	EBV and CMV	HSV and EBV	EBV and CMV	EBV	EBV and CMV
Lymphoproliferation	Yes	Yes	Yes	Yes	Yes	Yes
Lymphoma	No	No	No	No	No	Yes
Autoimmunity or inflammatory disease	SLE and secondary SS-like	SLE	SLE	SLE and enteropathy	SLE	SLE, AHA, and ITP MAS-like
Developmental retardation	No	Yes	Yes	No	Short stature	Growth retardation
References	This article	Wang et al. (17)	Wang et al. (17)	Wang et al. (17)	Li et al. (7)	Conti et al. (18)

APDS, activated phosphoinositide 3-kinase-δ syndrome; IgM, immunoglobulin M; IgG, immunoglobulin G; IgA, immunoglobulin A; EBV, Epstein–Barr virus; CMV, cytomegalovirus; HSV, herpes simplex virus; SLE, systemic lupus erythematosus; SS, Sjögren's syndrome; AHA, autoimmune hemolytic anemia; ITP, immune-mediated thrombocytopenia; MAS, macrophage activation syndrome; —, unknown.

Both patients with SLE and SS can present with hypergammaglobulinemia due to abnormal B-cell activation. However, hypergammaglobulinemia is more prominent in SS. In a cohort study, hypergammaglobulinemia presented in approximately half of the patients with primary SS and was associated with salivary gland damage (20). A previous study showed a positive correlation between a degree of lymphoid tissue infiltration in the labial salivary glands and hypergammaglobulinemia (21). The initial IgG level of our patient was significantly elevated and remained relatively high after treatment, which could be associated with recurrent parotitis, whereas the IgA and IgM levels were persistently higher than normal. Hyperglobulinemia is suggestive of B-cell hyperactivity and proliferation.

IVIG replacement therapy and antibiotic prophylaxis were not required for this patient because she did not present with a hyper-IgM phenotype or recurrent infections. Sirolimus, a target inhibitor of mTOR, is effective in treating benign lymphoproliferation but is less beneficial in treating APDS1-related-cytopenia and gastrointestinal diseases (13). Patients with autoimmune disorders require immunosuppressive therapy, which is beneficial for cytopenia. In this report, the patient's enlarged lymph nodes and spleen were completely retracted, and cytopenia and proteinuria were completely resolved after prednisone and sirolimus therapy. However, the recurrence of thrombocytopenia and occurrence of recurrent parotitis during prednisone tapering without discontinuation of sirolimus suggested that sirolimus was effective on lymphoproliferation and less effective on the autoimmune phenotype in our patient (Figure 4). Selective PI3K*δ* inhibitors, a promising new target treatment, are more attractive and useful for patients with APDS. It has been reported that leniolisib (CDZ173) was well tolerated and effectively improved clinical symptoms and laboratory parameters in APDS (22). It may also benefit SLE and SS due to both diseases being associated with high PI3K*δ* activity. Allogenic hematopoietic stem cell transplantation (HSCT) is a curative treatment; however, it carries a 10%–20% risk of mortality and vulnerability to renal complications continued post-HSCT (23, 24). We should choose the appropriate treatment according to the patient's specific situation.

Patients with APDS1 show significant clinical heterogeneity. In this case report, unlike the typical clinical presentation of APDS1, our patient presented predominantly with lymphoproliferation and an autoimmune phenotype instead of recurrent sinopulmonary infections or chronic viremia. In contrast to other reported patients with APDS1 with the SLE phenotype, our patient also mimicked a secondary SS-like phenotype, which is rare in APDS1. Our observations enrich the clinical phenotype of APDS1 and provide references for the diagnosis and therapy of patients with APDS1. However, a longer time is needed to observe the complete phenotype and long-term outcomes because APDS1 is a lifelong disease. In addition, the patient was found to have splenomegaly when she was 1 year old, which should be the clinical manifestation of this disease. However, her doctor did not pay enough attention to this important sign, which resulted in a delay in diagnosis. This reminds us of the importance of patient follow-up, especially in those patients with unexplained symptoms or signs. Early diagnosis can avoid or reduce complications.

## Data Availability

The original contributions presented in the study are included in the article/Supplementary Material, further inquiries can be directed to the corresponding author.
